# Missense mutations in *ITPR1* cause autosomal dominant congenital nonprogressive spinocerebellar ataxia

**DOI:** 10.1186/1750-1172-7-67

**Published:** 2012-09-17

**Authors:** Lijia Huang, Jodi Warman Chardon, Melissa T Carter, Kathie L Friend, Tracy E Dudding, Jeremy Schwartzentruber, Ruobing Zou, Peter W Schofield, Stuart Douglas, Dennis E Bulman, Kym M Boycott

**Affiliations:** 1 Children’s Hospital of Eastern Ontario Research Institute, University of Ottawa, Ottawa, ON, Canada; 2 Department of Genetics, Children’s Hospital of Eastern Ontario, Ottawa, ON, Canada; 3 Division of Clinical and Metabolic Genetics, Hospital for Sick Children, Toronto, ON, Canada; 4 Department of Genetic Medicine, Women’s and Children’s Hospital, SA Pathology, North Adelaide, Australia; 5 Hunter Genetics, Warratah, NSW, Australia; 6 University of Newcastle, Newcastle, NSW, Australia; 7 McGill University and Genome Quebec Innovation Centre, Montréal, QC, Canada; 8 Ottawa Hospital Research Institute, University of Ottawa, Ottawa, ON, Canada; 9 Centre for Translational Neuroscience and Mental Health, University of Newcastle, Newcastle, NSW, Australia; 10 Division of Neurology, Ottawa Hospital and University of Ottawa, Ottawa, ON, Canada

**Keywords:** Congenital nonprogressive spinocerebellar ataxia, Spinocerebellar ataxia type 29, Cerebellar atrophy, ITPR1, Gene identification

## Abstract

**Background:**

Congenital nonprogressive spinocerebellar ataxia is characterized by early gross motor delay, hypotonia, gait ataxia, mild dysarthria and dysmetria. The clinical presentation remains fairly stable and may be associated with cerebellar atrophy. To date, only a few families with autosomal dominant congenital nonprogressive spinocerebellar ataxia have been reported. Linkage to 3pter was demonstrated in one large Australian family and this locus was designated spinocerebellar ataxia type 29. The objective of this study is to describe an unreported Canadian family with autosomal dominant congenital nonprogressive spinocerebellar ataxia and to identify the underlying genetic causes in this family and the original Australian family.

**Methods and Results:**

Exome sequencing was performed for the Australian family, resulting in the identification of a heterozygous mutation in the *ITPR1* gene. For the Canadian family, genotyping with microsatellite markers and Sanger sequencing of *ITPR1* gene were performed; a heterozygous missense mutation in *ITPR1* was identified.

**Conclusions:**

*ITPR1* encodes inositol 1,4,5-trisphosphate receptor, type 1, a ligand-gated ion channel that mediates calcium release from the endoplasmic reticulum. Deletions of *ITPR1* are known to cause spinocerebellar ataxia type 15, a distinct and very slowly progressive form of cerebellar ataxia with onset in adulthood. Our study demonstrates for the first time that, in addition to spinocerebellar ataxia type 15, alteration of ITPR1 function can cause a distinct congenital nonprogressive ataxia; highlighting important clinical heterogeneity associated with the *ITPR1* gene and a significant role of the ITPR1-related pathway in the development and maintenance of the normal functions of the cerebellum.

## Background

The spinocerebellar ataxias (SCA) are a group of genetically heterogeneous disorders characterized by slowly progressive cerebellar dysfunction, including gait ataxia, dysarthria and limb dyscoordination. In addition, extra cerebellar features may be present, including cognitive impairment, ophthalmoplegia, pyramidal and extrapyramidal dysfunction [1]. In contrast to the more common autosomal dominant (AD) adult-onset SCAs, congenital nonprogressive spinocerebellar ataxia (CNPCA) is characterized by early motor delay and hypotonia, as well as other typical cerebellar features. Cognitive impairment may or may not be present. The clinical presentation remains stable throughout the life of an affected individual and some patients even report improvement in symptoms [2]. Cerebellar atrophy is often associated with CNPCA. Families with AD CNPCA, with and without cerebellar atrophy, have been reported approximately ten times since 1983 [2-10]. It is quite likely that AD CNPCA is a genetically heterogeneous condition [2,11]. Linkage to 3pter has been demonstrated in one large Australian family (SCA29), which overlaps with the locus for SCA15 [2]. SCA15 is a dominantly inherited slowly progressive cerebellar ataxia with mid-life onset; heterozygous *ITPR1* deletions spanning the entire or part of the gene are disease-causing [12-15].

The SCA29 locus overlaps with SCA15 making *ITPR1* an attractive candidate gene for AD CNPCA. Using a combination of exome and Sanger sequencing in the original SCA29 Australian family (Family A) and an unreported Canadian family (Family C), we identified two different missense mutations in *ITPR1* that segregate with the disease. Our study demonstrates that alterations in ITPR1 function are the cause of AD CNPCA (SCA29) and further highlights the important role of the ITPR1-related pathway in the development and maintenance of the cerebellum.

## M**ethods**

### Human subjects

We used standard methods to isolate genomic DNA from peripheral blood of the patients and their family members. Informed consent was obtained from all participating patients and families according to the Declaration of Helsinki and the studies were approved by the Research Ethics Boards of the Children’s Hospital of Eastern Ontario and Women’s and Children’s Hospital, North Adelaide.

### Genotyping

Genotyping was performed using DNA samples from all available members of Family C. The samples were amplified individually with 5 microsatellite markers (D3S4545, D3S1537, D3S1304, D3S3630, and D3S1297), spanning the 6.5 Mb region from 3p26.1 to 3q26.3. Haplotypes were constructed manually, and phase was assigned based on the minimum number of recombinants.

### Mutation analysis of *ITPR1*

Given the size of the mapped interval and number of genes, we used exome sequencing to analyze the critical region in Family A. Exome capture and massively parallel sequencing were performed at the McGill University and Genome Quebec Innovation Centre. In brief, the Agilent SureSelect 50 Mb All Exon Kit was used for target enrichment. Sequencing on Illumina HiSeq2000 platform generated >12Gbp of 100 bp paired-end data, giving ≥20x coverage of approximately 90% of the consensus coding sequence bases. Data analysis began with the removal of the adaptor sequences and trimming using the Fastx toolkit ( http://hannonlab.cshl.edu/fastx_toolkit/). Next, a custom script was used to select only those read pairs with both mates present for further alignment. Reads were aligned to human reference sequence (hg19) with BWA [16], and duplicate reads were marked using Picard ( http://picard.sourceforge.net/) and excluded from downstream analyses. Single nucleotide variants and short insertions and deletions were called using Samtools pileup ( http://samtools.sourceforge.net/) and varFilter [17] with the base alignment quality adjustment disabled, and were then quality-filtered to require at least 20% of reads supporting the variant call. Variants were annotated using both Annovar [18] and custom scripts to identify whether they affected protein coding sequence, and whether they were represented in dbSNP131, the 1000 genomes pilot release (Nov. 2010), or in approximately 120 exomes previously sequenced at the center. Sanger sequencing of the 58 exons and all associated exon/intron junctions of *ITPR1* in one affected individual from Family C was performed using standard methods.

The mutation identified in each family was confirmed to segregate as expected using Sanger sequencing. The primer pairs used for segregation analysis of Family A and Family C were: 5’ CTGGGTGTGAAAAACCTGCT 3’/ 5’ GCCCAGCTTAGATGCTCTGT 3’ and 5’ CATCAGGAAACATTGCTGCTT 3’/ 5’ AGCAGCACAAGGAAACGTAG 3’, respectively.

### Protein sequence alignment

Multiple sequence alignment was performed using clustalW2 ( http://www.ebi.ac.uk/Tools/msa/clustalw2/). Species that were compared were: *Homo sapiens* (Human; NP_001093422.2), *Pan troglodytes* (Chimpanzee; XP_003309637.1), *Mus musculus* (House mouse; NP_034715.3), *Rattus norvegicus* (Norway rat; NP_001007236.1), *Bos Taurus* (Cattle; NP_777266.1), *Gallus gallus* (Chicken; NP_001167530.1), *Xenopus laevis* (African clawed frog; NP_001084015.1), *Danio rerio* (Zebrafish; XP_001921194.2), *Metaseiulus occidentalis* (Western predatory mite; XP_003747783.1), *Apis mellifera* (Honey bee; XP_392236.4), and *Clonorchis sinensis* (Oriental liver fluke; GAA48211.1).

## Results

### Clinical features of Family C

**Figure 1:**
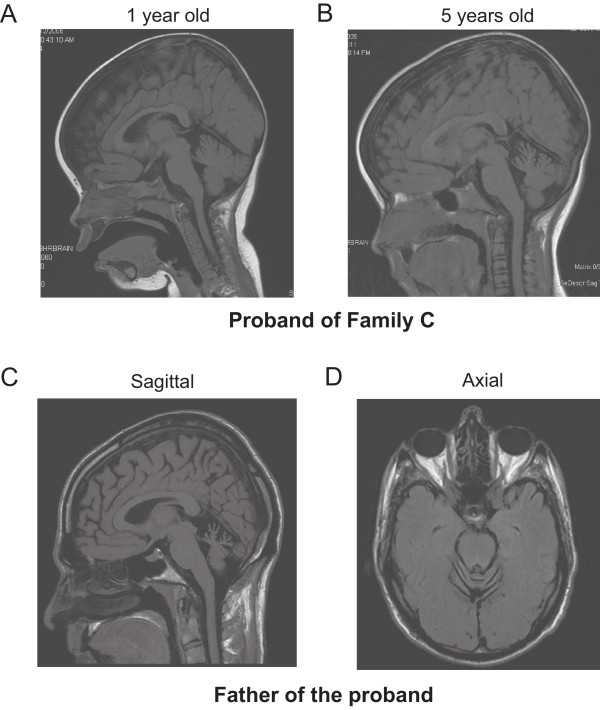
**Neuroimaging features of Family C. A**. T1 weighted sagittal MRI of the brain of the proband from Family C demonstrating mild cerebellar hypoplasia at 1 year of age.** B**. Demonstration of cerebellar atrophy in the proband at 5 years of age. **C**. and **D**. T1 weighted sagittal and axial MRI of the brain of the proband’s father at 45 years of age demonstrating diffuse cerebellar atrophy.

We identified a three-generation Canadian family with AD nonprogressive spinocerebellar ataxia. The proband was initially noted by her parents to have delayed sitting at 8 months of age. When examined in the Neurogenetics clinic at 28 months of age, her head circumference was 47.5 cm (25^th^ percentile). She demonstrated clear language delay; she was able to say 10 words, had 10 signs and followed simple commands. She had gaze-evoked nystagmus, hypotonia, truncal titubation and appendicular dysmetria and was not ambulating independently. Power, sensory, and deep tendon reflexes were normal. On reassessment at 3.5 years of age, we noted that while she was making developmental progress, she continued to exhibit cognitive delay, truncal titubation and limb ataxia. An MRI of the brain at 1 year of age demonstrated mild cerebellar vermal atrophy (Figure 1A), with progression of cerebellar vermal atrophy when the MRI was repeated at age 5 years (Figure 1B). She also developed complex partial seizures at age 5 years, and was found to have electrical status epilepticus of sleep on EEG. Her seizures and EEG improved with anti-epileptic medications. The proband has a clinically unaffected sister with normal development and no ataxia. The proband’s father, 45 years old, also demonstrated significantly delayed gross motor milestones, walking independently at 5 years of age. He described academic difficulties and repeated two primary grades, although he completed high school and some college courses. He is currently employed as a custodian. Physical examination demonstrated saccadic eye movements with end range nystagmus, dysarthria, gait and limb ataxia and intention tremor. Tone, power and deep tendon reflexes were normal and there were no sensory deficits. MRI of the brain at 45 years of age revealed diffuse cerebellar atrophy (Figure 1C and 1D). The proband’s paternal aunt ambulated independently at 4 years of age and repeated one primary grade. The proband’s paternal grandmother was similarly affected. All affected individuals had an unremarkable perinatal history. The proband’s mother had no evidence of ataxia and normal early development. However, she was diagnosed with Landau Kleffner syndrome at age 5 years, an acquired aphasia with epilepsy. She subsequently had full language recovery and remission of seizures.

### Genotyping analysis in Family C

**Figure 2:**
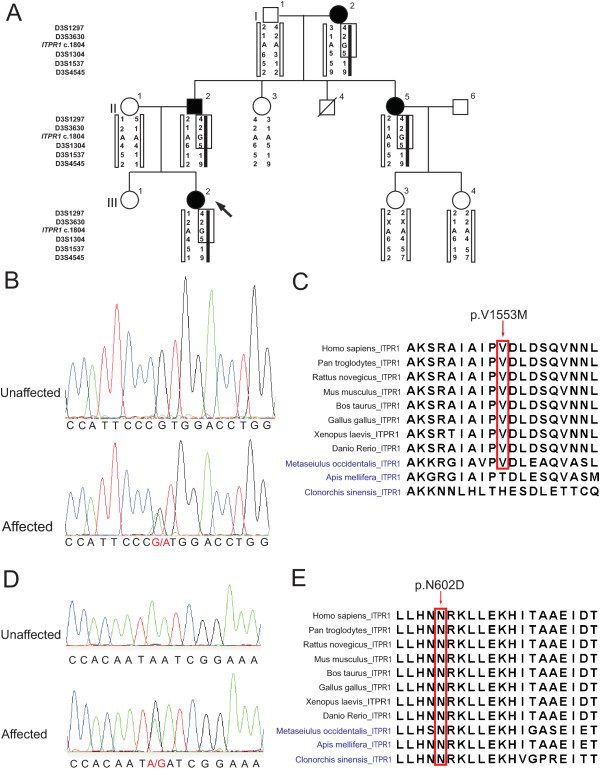
**Missense mutations in *ITPR1 *in two families with autosomal dominant congenital nonprogressive spinocerebellar ataxia. A**. Pedigree of Family C with AD CNPCA demonstrating segregation of the haplotype at 3pter with the disease (markers boxed in black). Affected individuals are represented by black symbols. A diagonal line indicates a deceased individual. Black arrow indicates the proband. **B.** Exome capture and massively parallel sequencing of III-6 from Family A identified a heterozygous mutation in *ITPR1* (NM_001099952.2:c.4657G >A; p.Val1553Met) which was confirmed by Sanger sequencing. Sequence traces from an unaffected (top) and an affected member (bottom) of Family A show the heterozygous mutation c.4657G>A (red) in the affected individual. **C**. Multiple sequence alignment of *Homo sapiens* ITPR1 against its orthologues from ten other species (vertebrates are labeled in black; non-chordates are labeled in blue) was performed using ClustalW. The mutated amino acid (residue 1553 in the human sequence) is boxed in red. **D**. Sequence traces from an unaffected (top) and affected member (bottom) of Family C show the heterozygous mutation c.1804A >G (red) in the affected individual. **E**. Multiple sequence alignment of *Homo sapiens* ITPR1 against its orthologues from ten other species (vertebrates are labeled in black; non-chordates are labeled in blue) was performed using ClustalW. The mutated amino acid (residue 602 in the human sequence) is boxed in red.

In our previously reported study of Family A with CNPCA [2], the disease locus was mapped to the terminal region of chromosome 3 (D3S1304 to 3pter, two-point lod score of 4.26), which contains the *ITPR1* gene [2]. Given the significant clinical overlap between Family C and the reported Australian family, we genotyped the affected and healthy family members of Family C with 5 microsatellite makers from 3pter. Haplotypes were constructed manually, and phase was assigned based on the minimum number of recombinants. The genotyping results indicated that all the affected family members shared a common haplotype in the region containing *ITPR1* and one unaffected individual (II-3) couldn’t be phased (Figure 2A). Chromosomal microarray analysis (Affymetrix 6.0 SNP array) of the proband was unrevealing; specifically there was no deletion of the *ITPR1* gene.

### Exome sequencing of the original SCA29 family (Family A)

Next, we wanted to determine if a mutation in *ITPR1*, or another gene in the critical region, is responsible for the SCA29 phenotype in the family from Australia [2]. We sequenced the exome of one affected individual from the family. The Agilent SureSelect 50 Mb All Exon Kit used in this study includes probes that target all 14 protein coding genes in the critical region. The mean read depth of these genes ranged from 52X to 205X. Nearly every base of the *ITPR1* gene had more than 50X coverage using this approach. After filtering out all the existent variants in dbSNP and the 1000 genomes pilot release, only one variant, a missense mutation in *ITPR1* (NM_001099952.2:c.4657G >A; p.Val1553Met), was identified within this critical region*.* In addition, this mutation was not found in 5379 exomes from the NHLBI Exome Sequencing Project. Sanger sequencing confirmed the segregation of the mutation with the disease in the family (Figure 2B and Additional file 1). The Valine residue at position 1553 is highly conserved among the vertebrates and certain non-chordates, such as *Metaseiulus occidentalis* (mites) (Figure 2C).

### Sequence analysis of *ITPR1* in Family C

Next, we PCR amplified and Sanger sequenced all 58 exons and the exon/intron boundaries of the *ITPR1* gene in one affected individual from Family C and identified a missense mutation (NM_001099952.2:c.1804A >G; p.Asn602Asp) (Figure 2D), which segregated with the disease phenotype in the family. This mutation was not found in 136 controls of European descent, the 1000 genomes project, or the NHLBI Exome Sequencing Project (5379 exomes). Moreover, the Asparagine at position 602 is a highly conserved residue among both vertebrates and non-chordates (Figure 2E).

## Discussion

We describe two families with AD congenital nonprogressive spinocerebellar ataxia caused by missense mutations in *ITPR1*, demonstrating for the first time clinical heterogeneity associated with alterations of this gene. Cerebellar atrophy has been identified as an early feature of this disorder and was observed in the proband of Family C at 28 months. Interestingly, serial imaging of the proband from this family demonstrates that, at least in this case, the cerebellar atrophy continues to progress (Figure 1A and 1B). Given that she continues to slowly make developmental gains we would classify her presentation as a clinically nonprogressive ataxia. In Family A, five members reported improvement in ataxia [2]. Amelioration in ataxia has been reported in an additional series of patients with CNPCA [3,7,8,19]. The mechanism by which patients demonstrate an improvement in ataxia is unknown but may result from early adaptation to the cerebellar dysfunction, particularly in instances where the atrophy is nonprogressive. In addition to cerebellar ataxia and cognitive impairment, the proband of Family C had epilepsy. There have been reports of congenital cerebellar dysfunction associated with electrical status of slow wave sleep, as seen in the proband of this study [20]. It is unclear if the occurrence of seizures in the proband is related to the *ITPR1* mutation; this possible association will be better understood as further patients are identified.

SCA15 and SCA29 are distinctly different disorders; clinical features distinguishing SCA15 from SCA29 include the age of onset (adulthood versus congenital), clinical progression (progressive vs static) and the occurrence of mild intellectual disability (present in SCA29). SCA15 has been reported to be the most common nontrinucleotide repeat SCA in central Europe accounting for 8.9% of SCA families negative for common SCA repeat expansions [21]. Deletions of the *ITPR1* gene are the underlying genetic cause of almost all cases of SCA15 [12-15,21]. So far, only one family with adult-onset SCA has been reported to harbor a missense mutation in *ITPR1*[13] and the Ca2+ release properties of this mutant ITPR1 are comparable with wild type ITPR1: therefore, functional pathogenicity of this change has not been established or there is another mechanism for the disease process [22]. The mechanism by which different mutations in *ITPR1* can cause two different phenotypes, SCA15 and SCA29, is unclear.

The *ITPR1* gene encodes type 1 inositol 1,4,5-trisphosphate (IP_3_) receptor, a ligand-gated Ca^2+^ channel on the endoplasmic reticulum membrane. Upon binding to IP_3_, ITPR1 channels release Ca^2+^ into the cytoplasm producing complex Ca^2+^ signals that are involved in various cellular processes [23]. ITPR1 functions as a homotetramer or heterotetramer with type 2 or type 3 inositol 1,4,5-trisphosphate receptor, and ITPR1 is the most abundant isoform in the central nervous system, particularly enriched in cerebellar Purkinje cells [23]. In almost all SCA15 cases, partial or complete deletion of *ITPR1* gene suggests that ITPR1 haploinsufficiency is the predominant mechanism. The missense mutations in *ITPR1* that cause SCA29 are localized to the coupling/regulatory domain of ITPR1. The coupling/regulatory domain, containing protein phosphorylation sites, a proteolytic cleavage site, ATP binding sites, and binding sites for numerous proteins, modulates ITPR1 function by integrating other signaling pathways [24]. Asn602 is located in the IRBIT binding domain and Val1553 is located within the binding domain for CA8. Interestingly, both IRBIT and CA8 are competitors of IP_3_ for binding to ITPR1. Mutations in *CA8* cause a recessive form of congenital ataxia associated with mild intellectual disability [25]. The only known function of CA8 is that it inhibits IP_3_ binding to ITPR1 [26], therefore ITPR1 may be more sensitive to IP_3_ in *CA8*-deficient patients and the disease would result from dysregulation of the channel. Thus, it is likely that the two SCA29-causing missense mutations reported here affect the normal regulation of ITPR1.

Dysfunction of the ITPR1-mediated Ca^2+^ signaling pathway is implicated in the development of several types of late-onset ataxia in addition to SCA15. The expression of *Itpr1,* among other neuronal genes, is downregulated in the transgenetic SCA1 mouse models expressing mutant ataxin-1 [27]. Mutant ataxin-2 and ataxin-3, but not the wild-type proteins, can interact with ITPR1 and sensitize its IP_3_-induced Ca^2+^ release causing disruption of calcium signalling in the mutant neurons [28,29]. *Itpr1* also plays an important role in embryonic development; the majority of *Itpr1* null mice die prenatally and those which survive to birth display severe ataxia and epilepsy [30]. Similar symptoms were seen in two spontaneous *Itpr1* mutant mouse models [12,31]. Interestingly, the heterozygous null mice grow normally but present with late-onset motor discoordination [32]. These results implicate an essential role of ITPR1-dependent signaling in both cerebellar development and maintenance.

## Conclusions

In summary, our study demonstrates that missense mutations in *ITPR1* cause AD congenital nonprogressive spinocerebellar ataxia (SCA29) and further emphasizes the importance of the ITPR1-dependent pathway in the development and maintenance of the normal functions of cerebellum. This finding expands the phenotypic spectrum caused by *ITPR1* mutations and will facilitate molecular diagnosis for patients with congenital nonprogressive spinocerebellar ataxia.

## Abbreviations

AD: Autosomal dominant; CNPCA: Congenital nonprogressive spinocerebellar ataxia; IP_3_: Inositol 1, 4, 5-trisphosphate; SCA: Spinocerebellar ataxia.

## Competing interests

The authors declare that they have no competing interests.

## Authors’ contributions

LH designed the study, performed the genotyping and sequence analysis, and wrote the manuscript. JW contributed to the analysis of the clinical and MRI data and wrote the manuscript. MTC contributed to the collection of the clinical data for Family C. KLF, TED and PWS contributed to the collection of the clinical data of Family A. JS carried out the analysis of the next-generation sequencing data. RZ performed genotyping and Sanger sequencing studies. SD performed genotyping analysis. DEB supervised the molecular data analysis. KMB designed and coordinated the study, contributed to the acquisition and analysis of the clinical and MRI data and wrote the manuscript. All authors read and approved the final manuscript.

## Supplementary Material

Additional file 1 Pedigree of Family A (original SCA29 family). Click here for file

## References

[B1] Harding AE Classification of the hereditary ataxias and paraplegias Lancet 1983 1 1151 1155 6133167 10.1016/s0140-6736(83)92879-9

[B2] Dudding TE Friend K Schofield PW Lee S Wilkinson IA Richards RI Autosomal dominant congenital non-progressive ataxia overlaps with the SCA15 locus Neurology 2004 63 2288 2292 10.1212/01.WNL.0000147299.80872.D1 15623688

[B3] Tomiwa K Baraitser M Wilson J Dominantly inherited congenital cerebellar ataxia with atrophy of the vermis Pediatr Neurol 1987 3 360 362 10.1016/0887-8994(87)90008-7 3334022

[B4] Kattah JC Kolsky MP Guy J O'Doherty D Primary position vertical nystagmus and cerebellar ataxia Arch Neurol 1983 40 310 314 10.1001/archneur.1983.04050050078012 6847426

[B5] Fenichel GM Phillips JA Familial aplasia of the cerebellar vermis. Possible X-linked dominant inheritance Arch Neurol 1989 46 582 583 10.1001/archneur.1989.00520410118036 2469415

[B6] Furman JM Baloh RW Chugani H Waluch V Bradley WG Infantile cerebellar atrophy Ann Neurol 1985 17 399 402 10.1002/ana.410170417 4004161

[B7] Rivier F Echenne B Dominantly inherited hypoplasia of the vermis Neuropediatrics 1992 23 206 208 10.1055/s-2008-1071342 1407388

[B8] Imamura S Tachi N Oya K Dominantly inherited early-onset non-progressive cerebellar ataxia syndrome Brain Dev 1993 15 372 376 10.1016/0387-7604(93)90124-Q 8279653

[B9] Kornberg AJ Shield LK An extended phenotype of an early-onset inherited nonprogressive cerebellar ataxia syndrome J Child Neurol 1991 6 20 23 10.1177/088307389100600104 2002196

[B10] Titomanlio L Pierri NB Romano A Imperati F Borrelli M Barletta V Diano AA Castaldo I Santoro L Del Giudice E Cerebellar vermis aplasia: patient report and exclusion of the candidate genes EN2 and ZIC1 Am J Med Genet A 2005 136 198 200 15940696 10.1002/ajmg.a.30795

[B11] Jen JC Lee H Cha YH Nelson SF Baloh RW Genetic heterogeneity of autosomal dominant nonprogressive congenital ataxia Neurology 2006 67 1704 1706 10.1212/01.wnl.0000242705.06416.6a 17101914

[B12] van de Leemput J Chandran J Knight MA Holtzclaw LA Scholz S Cookson MR Houlden H Gwinn-Hardy K Fung HC Lin X Hernandez D Simon-Sanchez J Wood NW Giunti P Rafferty I Hardy J Storey E Gardner RJ Forrest SM Fisher EM Russell JT Cai H Singleton AB Deletion at ITPR1 underlies ataxia in mice and spinocerebellar ataxia 15 in humans PLoS Genet 2007 3 e108 10.1371/journal.pgen.0030108 17590087 PMC1892049

[B13] Hara K Shiga A Nozaki H Mitsui J Takahashi Y Ishiguro H Yomono H Kurisaki H Goto J Ikeuchi T Tsuji S Nishizawa M Onodera O Total deletion and a missense mutation of ITPR1 in Japanese SCA15 families Neurology 2008 71 547 551 10.1212/01.wnl.0000311277.71046.a0 18579805

[B14] Iwaki A Kawano Y Miura S Shibata H Matsuse D Li W Furuya H Ohyagi Y Taniwaki T Kira J Fukumaki Y Heterozygous deletion ofITPR1, but not SUMF1, in spinocerebellar ataxia type 16 J Med Genet 2008 45 32 35 17932120 10.1136/jmg.2007.053942

[B15] Ganesamoorthy D Bruno DL Schoumans J Storey E Delatycki MB Zhu D Wei MK Nicholson GA McKinlay Gardner RJ Slater HR Development of a multiplex ligation-dependent probe amplification assay for diagnosis and estimation of the frequency of spinocerebellar ataxia type 15 Clin Chem 2009 55 1415 1418 10.1373/clinchem.2009.124958 19423733

[B16] Li H Durbin R Fast and accurate short read alignment with Burrows-Wheeler transform Bioinformatics 2009 25 1754 1760 10.1093/bioinformatics/btp324 19451168 PMC2705234

[B17] Li H Handsaker B Wysoker A Fennell T Ruan J Homer N Marth G Abecasis G Durbin R The Sequence Alignment/Map format and SAMtools Bioinformatics 2009 25 2078 2079 10.1093/bioinformatics/btp352 19505943 PMC2723002

[B18] Wang K Li M Hakonarson H ANNOVAR: functional annotation of genetic variants from high-throughput sequencing data Nucleic Acids Res 2010 38 e164 10.1093/nar/gkq603 20601685 PMC2938201

[B19] Tsao JW Neal J Apse K Stephan MJ Dobyns WB Hill RS Walsh CA Sheen VL Cerebellar ataxia with progressive improvement Arch Neurol 2006 63 594 597 10.1001/archneur.63.4.594 16606775

[B20] Parmeggiani A Posar A Scaduto MC Cerebellar hypoplasia, continuous spike-waves during sleep, and neuropsychological and behavioral disorders J Child Neurol 2008 23 1472 1476 10.1177/0883073808319077 19073855

[B21] Synofzik M Beetz C Bauer C Bonin M Sanchez-Ferrero E Schmitz-Hubsch T Wullner U Nagele T Riess O Schols L Bauer P Spinocerebellar ataxia type 15: diagnostic assessment, frequency, and phenotypic features J Med Genet 2011 48 407 412 10.1136/jmg.2010.087023 21367767

[B22] Yamazaki H Nozaki H Onodera O Michikawa T Nishizawa M Mikoshiba K Functional characterization of the P1059L mutation in the inositol 1,4,5-trisphosphate receptor type 1 identified in a Japanese SCA15 family Biochem Biophys Res Commun 2011 410 754 758 10.1016/j.bbrc.2011.06.043 21689634

[B23] Foskett JK White C Cheung KH Mak DO Inositol trisphosphate receptor Ca2+ release channels Physiol Rev 2007 87 593 658 10.1152/physrev.00035.2006 17429043 PMC2901638

[B24] Foskett JK Inositol trisphosphate receptor Ca2+ release channels in neurological diseases Pflugers Arch 2010 460 481 494 10.1007/s00424-010-0826-0 20383523 PMC2893360

[B25] Turkmen S Guo G Garshasbi M Hoffmann K Alshalah AJ Mischung C Kuss A Humphrey N Mundlos S Robinson PN CA8 mutations cause a novel syndrome characterized by ataxia and mild mental retardation with predisposition to quadrupedal gait PLoS Genet 2009 5 e1000487 10.1371/journal.pgen.1000487 19461874 PMC2677160

[B26] Hirota J Ando H Hamada K Mikoshiba K Carbonic anhydrase-related protein is a novel binding protein for inositol 1,4,5-trisphosphate receptor type 1 Biochem J 2003 372 435 441 10.1042/BJ20030110 12611586 PMC1223404

[B27] Lin X Antalffy B Kang D Orr HT Zoghbi HY Polyglutamine expansion down-regulates specific neuronal genes before pathologic changes in SCA1 Nat Neurosci 2000 3 157 163 10.1038/72101 10649571

[B28] Chen X Tang TS Tu H Nelson O Pook M Hammer R Nukina N Bezprozvanny I Deranged calcium signaling and neurodegeneration in spinocerebellar ataxia type 3 J Neurosci 2008 28 12713 12724 10.1523/JNEUROSCI.3909-08.2008 19036964 PMC2663415

[B29] Liu J Tang TS Tu H Nelson O Herndon E Huynh DP Pulst SM Bezprozvanny I Deranged calcium signaling and neurodegeneration in spinocerebellar ataxia type 2 J Neurosci 2009 29 9148 9162 10.1523/JNEUROSCI.0660-09.2009 19625506 PMC2749883

[B30] Matsumoto M Nakagawa T Inoue T Nagata E Tanaka K Takano H Minowa O Kuno J Sakakibara S Yamada M Yoneshima H Miyawaki A Fukuuchi Y Furuichi T Okano H Mikoshiba K Noda T Ataxia and epileptic seizures in mice lacking type 1 inositol 1,4,5-trisphosphate receptor Nature 1996 379 168 171 10.1038/379168a0 8538767

[B31] Street VA Bosma MM Demas VP Regan MR Lin DD Robinson LC Agnew WS Tempel BL The type 1 inositol 1,4,5-trisphosphate receptor gene is altered in the opisthotonos mouse J Neurosci 1997 17 635 645 8987786 10.1523/JNEUROSCI.17-02-00635.1997 PMC6573232

[B32] Ogura H Matsumoto M Mikoshiba K Motor discoordination in mutant mice heterozygous for the type 1 inositol 1,4,5-trisphosphate receptor Behav Brain Res 2001 122 215 219 10.1016/S0166-4328(01)00187-5 11334652

