# Correction to: Missense mutations in *ITPR1* cause autosomal dominant congenital nonprogressive spinocerebellar ataxia

**DOI:** 10.1186/s13023-022-02297-7

**Published:** 2022-03-29

**Authors:** Lijia Huang, Jodi Warman-Chardon, Melissa T. Carter, Kathie L. Friend, Tracy E. Dudding, Jeremy Schwartzentruber, Ruobing Zou, Peter W. Schofield, Stuart Douglas, Dennis E. Bulman, Kym M. Boycott

**Affiliations:** 1 Children’s Hospital of Eastern Ontario Research Institute, University of Ottawa, Ottawa, ON Canada; 2 Department of Genetics, Children’s Hospital of Eastern Ontario, Ottawa, ON Canada; 3 Division of Clinical and Metabolic Genetics, Hospital for Sick Children, Toronto, ON Canada; 4 Department of Genetic Medicine, Women’s and Children’s Hospital, SA Pathology, North Adelaide, Australia; 5 Hunter Genetics, Warratah, NSW Australia; 6 University of Newcastle, Newcastle, NSW Australia; 7 McGill University and Genome Quebec Innovation Centre, Montréal, QC Canada; 8 Ottawa Hospital Research Institute, University of Ottawa, Ottawa, ON Canada; 9 Centre for Translational Neuroscience and Mental Health, University of Newcastle, Newcastle, NSW Australia; 10 Division of Neurology, Ottawa Hospital and University of Ottawa, Ottawa, ON Canada

## Correction to: Orphanet Journal of Rare Diseases 2012, 7:67 https://doi.org/10.1186/1750-1172-7-67

The original article [1] unfortunately contained an error to the second author’s name. The article erroneously presented “Jodi” and “Warman” as given names, and “Chardon” as family name. Instead, the author’s given name is “Jodi” and family name is “Warman-Chardon”.

The correct name is included in the author list of this Correction and has already been updated in the original article.
