# Supplementary material for: Missense mutations in ITPR1 cause autosomal dominant congenital nonprogressive spinocerebellar ataxia
Source: Orphanet J Rare Dis. 2012 Sep 17;7:67. doi: 10.1186/1750-1172-7-67 (PMC3545966; doi:10.1186/1750-1172-7-67)
Supplement: Additional file 1 — Pedigree of Family A (original SCA29 family). [file 1750-1172-7-67-S1.doc]

**Additional file 1**

**Title: Pedigree of Family A (original SCA29 family)**.

**Description:** Figure S1. Family relationships and segregation of the mutation (NM_001099952.2:c.4657G>A; p.Val1553Met) in *ITPR1* are shown.

File format: PDF
